# Development of Cobalt-Binding Peptide Chelate from Human Serum Albumin: Cobalt-Binding Properties and Stability

**DOI:** 10.3390/ijms23020719

**Published:** 2022-01-10

**Authors:** Yeonje Cho, Armin Mirzapour-Kouhdasht, Hyosuk Yun, Jeong Hoon Park, Hye Jung Min, Chul Won Lee

**Affiliations:** 1 Department of Chemistry, Chonnam National University, Gwangju 61186, Korea; yeonje20@gmail.com (Y.C.); armin.mirzapourkouhdahst@ucd.ie (A.M.-K.); 5300747yun@hanmail.net (H.Y.); 2 School of Agriculture and Food Science, University College Dublin, Belfield, D04 V1W8 Dublin, Ireland; 3 Accelerator Radioisotope Development Laboratory, Korea Atomic Energy Research Institute, Jeongeup-si 56212, Jeollabuk-do, Korea; parkjh@kaeri.re.kr; 4 Department of Cosmetic Science, Kwangju Women’s University, Gwangju 62396, Korea; sarock@kwu.ac.kr

**Keywords:** peptides, cobalt binding, structure, stability

## Abstract

Radioactive isotopes are used as drugs or contrast agents in the medical field after being conjugated with chelates such as DOTA, NOTA, DTPA, TETA, CyDTA, TRITA, and DPDP. The N-terminal sequence of human serum albumin (HSA) is known as a metal binding site, such as for Co^2+^, Cu^2+^, and Ni^2+^. For this study, we designed and synthesized wAlb12 peptide from the N-terminal region of HSA, which can bind to cobalt, to develop a peptide-based chelate. The wAlb12 with a random coil structure tightly binds to the Co(II) ion. Moreover, the binding property of wAlb12 toward Co(II) was confirmed using various spectroscopic experiments. To identify the binding site of wAlb12, the analogs were synthesized by alanine scanning mutagenesis. Among them, H3A and Ac-wAlb12 did not bind to Co(II). The analysis of the binding regions confirmed that the His3 and α-amino group of the N-terminal region are important for Co(II) binding. The wAlb12 bound to Co(II) with *K*_d_ of 75 μM determined by isothermal titration calorimetry when analyzed by a single-site binding model. For the use of wAlb12 as a chelate in humans, its cytotoxicity and stability were investigated. Trypsin stability showed that the wAlb12 − Co(II) complex was more stable than wAlb12 alone. Furthermore, the cell viability analysis showed wAlb12 and wAlb12 + Co(II) to be non-toxic to the Raw 264.7 and HEK 293T cell lines. Therefore, a hot radioactive isotope such as cobalt-57 will have the same effect as a stable isotope cobalt. Accordingly, we expect wAlb12 to be used as a peptide chelate that binds with radioactive isotopes.

## 1. Introduction

**Figure 1:**
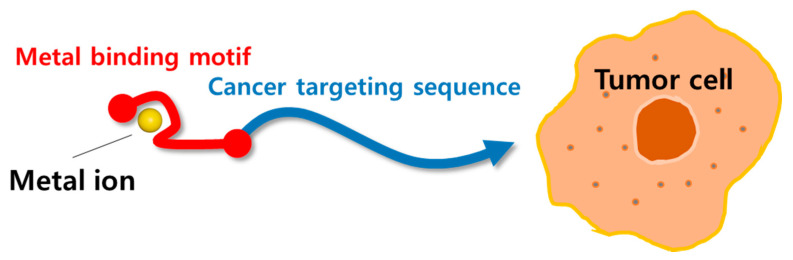
Schematic diagram of peptide-based metal chelate for cancer diagnosis and therapy.

Radioactive isotopes are widely used for diagnosis and therapy in nuclear medicine during PET, CT, and X-ray examinations, and their importance for this purpose is rapidly increasing. Several radioisotopes are already used in medicine. For instance, radioactive isotope technetium-99m is universally applied for imaging the human brain, heart, lungs, liver, and bones [1]. Iodine-131 is also known to be effective against hyperthyroidism and thyroid tumors [2]. In addition, cobalt and copper have been used for the diagnosis and therapy of various cancers in humans. ^60^Co releases powerful gamma radiation, which is used in radiotherapy, and ^64^Cu is utilized as the radiotracer for cancer detection with bombesin peptide analogs [3,4,5]. Moreover, ^68^Ga is used for the PET/CT scanning diagnosis of various types of neuroendocrine cancers, and ^89^Zr is used for SPECT/CT for human organ imaging, including that of the liver, lungs, heart, brain, and bones [6,7,8]. Chelating agents for therapeutic use generally have a spiral structure and strong binding properties with metal ions [9]. Chelating ligands usually include chemical structures, such as amines, carboxyl acids, esters, aromatic isothiocyanates, anhydrides, epoxides, aldehydes, and azides [9,10]. The use of these chelating agents with metal ions prevents dissociation owing to heat. Chelators also exhibit kinetic inactivation in vivo [9]. In medicine, for drugs or contrast media, radioactive isotopes are used following conjugation with chelators such as DOTA, NOTA, DTPA, TETA, CyDTA, TRITA, and DPDP [10]. Chelating agents bound to radioisotopes are conjugated with ligands such as peptides, proteins, organic chemicals, and nanoparticles for use as drugs and as transporting agents with a linker. For example, ^131^I is conjugated with a DOTA chelating agent and ^89^Zr is conjugated with an oxalate or deferoxamine chelating agent [2,7,8]. Additionally, ^68^Ga is used in conjugation with the NOTA chelating agent as a bone-seeking agent for PET imaging [6]. Thus, chelating agents have been used in close association with radioisotopes as diagnostic and therapeutic agents for various human diseases. Metal-binding proteins constitute approximately one-third of all proteins [11]. The commonly geometric metal binding structure of proteins and peptides is related to the imidazole group of histidine, sulfhydryl group of cysteine, and carboxyl groups of aspartic and glutamic acid [11,12,13,14]. These metalloproteins, which include hemoglobin, albumin, calmodulin, and rubredoxin, have side groups involved in metal binding. During protein folding, the amino acids involved in metal binding are spatially adjacent to each other, and when the metal is inserted between them, the binding is stable [11,13]. In peptides, sequences involved in metal binding exhibit the same metal-binding protein protocol. These peptides have structural similarities, such as the HxH, CxxC, CxH, MxC, LxHxD, or glutathione (ExCxG) motif [11,12,13]. These common amino acids form the spatial structure that mediates binding to metal ions. Moreover, many metal-binding peptides are now known, and their binding properties and structural positions in relation to metals are being studied [15,16,17,18,19,20,21,22,23,24]. Peptides can be highly selective and effective signaling molecules that bind to specific cell surface receptors, such as cancer cell receptors or ion channels, and exhibit various activities in cells [25]. These properties of peptides not only aid in the specific killing of cancer cells but also help deliver drugs to the target location, demonstrating the benefits of biodegradation in the body without accumulation [14,25]. These peptides also have a high tolerability, a predictable metabolism, and unlimited applicability in other fields [26]. Furthermore, peptide drugs are commonly associated with lower production intricacy than protein-based biochemical drugs, and the cost of peptide production is low because of the small size of the molecules [14,25]. Therefore, peptide-based medicines are now widely developed and commercially available. Using these advantages of peptides along with their ability to bind metals, we designed a peptide-based metal chelate that binds radioisotopes instead of chemical ligands for application in targeted therapy (Figure 1). The amino terminal Cu(II)- and Ni(II)-binding (ATCUN) motif is found in the N-terminus of many naturally occurring peptides and proteins, such as albumins, neuromedin C, histatin 5, and copper transporter 1. The sequence of ATCUN motif is composed of a His residue at third position with two arbitrary amino acids at the first and second positions (NH_2_-X^1^X^2^H^3^, where X is an arbitrary amino acid). The free N-terminal amino group is also critical for the metal binding property of ATCUN motif [27,28,29,30]. The N-terminal sequence of human serum albumin (HSA) is a metal binding site for Co^2+^, Cu^2+^, and Ni^2+^. The N-terminal four residues (Asp-Ala-His-Lys) of HSA binds the Co^2+^ and Ni^2+^. It provides four nitrogen ligands: an imidazole nitrogen of His3, the N-terminal amino group, and two deprotonated backbone amide nitrogens. In addition, the carboxylate side chain of Asp1 is also implicated in metal binding, giving an overall penta-coordinate site, although this residue does not appear to be crucial for the high affinity Cu^2+^ and Ni^2+^ binding. In particular, the first three amino acids, Asp-Ala-His, are essential for strong binding of Co^2+^ [28,31]. In this study, we designed and synthesized wAlb12 (DAHKSEVAHRFK), a peptide derived from the N-terminal region of HSA, that can tightly bind Co(II). We confirmed the binding site, properties, intensities, and stabilities for the binding of Co(II) to wAlb12 using various biochemical and biophysical experiments.

## 2. Results and Discussion

### 2.1. Design and Synthesis of Co(II)-Binding Peptides

We first designed and synthesized the peptides with the ability to bind to Co(II) (Table 1). The wAlb12 peptides were derived from the N-terminal sequence of HSA with a binding ability to Co(II). Moreover, a poly(his)8 composed of eight histidines was designed and synthesized for its high affinity binding to Co(II). SMAP18 (G2–G13) derived from sheep myeloid antimicrobial peptide-18 was used as the negative control to compare binding with Co(II) between the peptides. In addition, we performed alanine scanning of wAlb12 to identify the Co(II) binding site. All of the peptides were purified by preparative RP-HPLC. The molecular weight of all of the prepared peptides was confirmed by LC-MS analysis (Figures S1 and S2). The development of peptide-based chelates is known to have some advantages, such as stronger binding ability, higher bioavailability, and stability under various pH and temperature conditions, as well as low cytotoxicity [32,33,34]. Thus, we designed and synthesized peptides that can function as metal chelators with good safety, stability, and binding properties. The Co(II)-binding property of the synthesized wAlb12 was investigated using UV, CD, NMR, and LC-MS analyses. To prepare the sample of peptide + Co(II), we conducted self-binding of Co(II) in a buffer with a neutral pH of 7.4. Then, poly(his)8 was synthesized to bind to the metal with a histidine residue related to the metal binding sequence. In a slightly contrasting manner, the poly(his)8 + Co(II) lead to the formation of a yellow crystal deposit because the strong interaction with Co(II) resulted in aggregation and crystallization. Accordingly, we investigated the Co(II)-binding property of wAlb12 and SMAP18 (G2–G13).

### 2.2. UV Spectrophotometry

**Figure 2:**
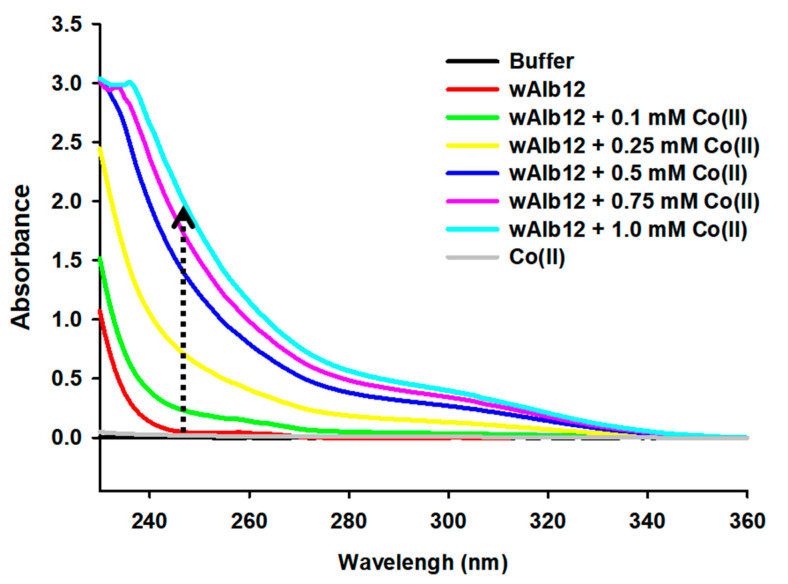
Change in the UV spectrum of 0.5 mM wAlb12 by Co(II) binding at wavelengths between 230–360 nm.

**Figure 3:**
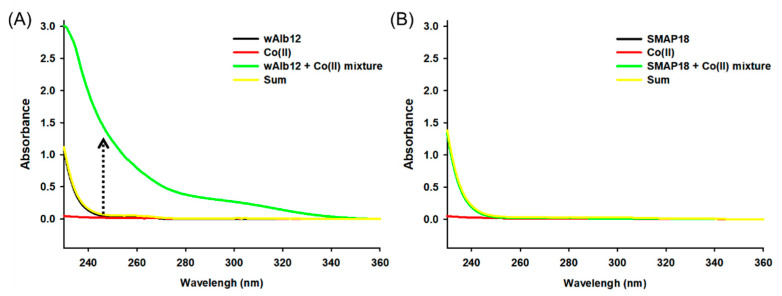
Change in the UV spectrum of peptides by Co(II) binding. wAlb12 (**A**) and SMAP18 (G2–G13) (**B**) at 230–360 nm.

First, to identify the UV spectral change due to binding with Co(II), the wAlb12–Co(II) binding was confirmed using UV spectrophotometry (Figure 2). The results showed increased UV intensity between the wavelengths 230 and 340 nm with an increase in CoCl_2_ concentrations. The baseline measured the 50 mM sodium phosphate containing 10 mM sodium chloride buffer solution. The UV intensity of 1 mM CoCl_2_ showed a spectrum similar to that shown by the baseline. wAlb12 showed a slightly increased UV intensity at 230–360 nm wavelengths. As the CoCl_2_ concentration increased, the UV intensity of wAlb12 + Co(II) also gradually increased at 230–280 nm. These results revealed that the UV intensity of wAlb12 + Co(II) was increased compared to that of wAlb12 and CoCl_2_ because of the Co(II) bound to wAlb12. Next, to evaluate Co(II) binding to specific peptides, the wAlb12 and SMAP18 (G2–G13) peptides were compared. The UV intensities of 0.5 mM wAlb12, CoCl_2_, and buffer were measured (Figure 3). Thereafter, the sum of the wAlb12 and Co(II) values was obtained by the addition of the UV intensity values of CoCl_2_ and wAlb12. The wAlb12 + Co(II) mixture represented the UV intensity value of wAlb12-bound Co(II). In these spectra, the wAlb12 + Co(II) mixture showed a higher value than the sum of the values obtained for wAlb12 and Co(II). In the wavelength range of 230–360 nm, wAlb12 showed a small peak at 230–240 nm. The UV intensity of CoCl_2_ was similar to that of the baseline (measured with the buffer solution). In addition, the sum of the values of wAlb12 and Co(II) was similar to the value obtained for wAlb12. Meanwhile, the wAlb12 + Co(II) mixture showed a value three times higher than that of wAlb12 alone and that of the sum of the wAlb12 and Co(II) values (Figure 3A). On the other hand, the UV intensity of SMAP18 (G2–G13) was not changed (Figure 3B). These results indicate that SMAP18 (G2–G13) does not bind to Co(II), while wAlb12 does. We considered SMAP18 (G2–G13) as a comparison control peptide that has no metal binding sequences, such as histidine, aspartic acid, tryptophan, glutamic acid, and cysteine [11,12]. Thus, we predicted and confirmed that the Co(II) does not bind to SMAP18 (G2–G13). The results of this study show the selective binding of Co(II) to wAlb12.

### 2.3. Monitoring of Co(II) Binding to wAlb12 by CD Spectroscopy

**Figure 4:**
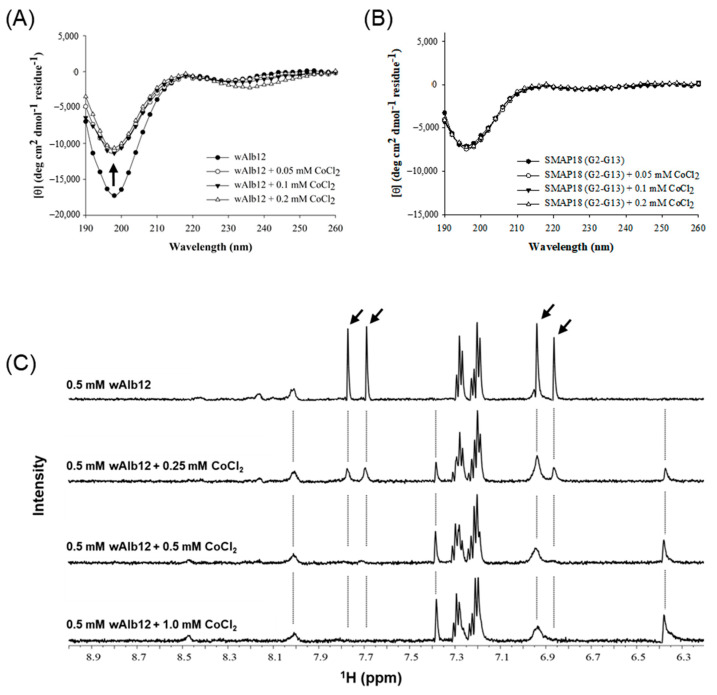
The CD and NMR spectral changes of peptides by Co(II) binding. The CD spectra of wAlb12 (**A**) and SMAP18 (G2-G13) (**B**). (**C**) 1D proton NMR spectral changes of the wAlb12 by adding of CoCl_2_. Arrows indicates that the peaks were largely broadened out by adding of CoCl_2_.

The change in the wAlb12 structure by Co(II) binding was investigated using CD spectroscopy. The CD spectra indicated that the peptide exhibited a random coil structure at a concentration of 0.1 mM in a buffer containing 50 mM sodium phosphate and 10 mM sodium chloride (Figure 4A). The CoCl_2_ to peptide concentration ratios were 1:0.5, 1:1, and 1:2. For wAlb12, when the Co(II) was added, the CD spectrum at 200 nm showed a sharp increase in values. In contrast, the random coil form of the SMAP18 (G2–G13) peptide showed the same spectra in the absence and presence of Co(II) (Figure 4B). Accordingly, Co(II) was not bound to SMAP18 (G2–G13) and the CD spectra were not changed.

### 2.4. NMR Spectroscopy

The proton NMR technique was performed to investigate the binding properties of Co(II) on the wAlb12 peptide (Figure 4C). The chemical shift patterns of 0.5 mM wAlb12 showed relatively high peaks of approximately 6.9, 7.3, and 7.7 ppm. When 0.25 mM Co(II) was added to wAlb12, the peaks of wAlb12 gradually and inversely decreased at 6.9 and 7.7 ppm. With an increase in Co(II) concentration up to 0.5 mM (at 1:1 ratio), the peaks almost disappeared, whereas the peaks of 6.4 and 7.4 ppm appeared starting with 0.25 mM Co(II). Progressively increasing new peaks appeared at 6.4 and 7.4 ppm as the Co(II) concentration increased, but the peaks of approximately 7.3 ppm showed little change. These results indicate that the peaks of 6.9 and 7.7 ppm are related to the binding site for the paramagnetic Co(II) ion.

### 2.5. Mass Spectroscopy

**Figure 5:**
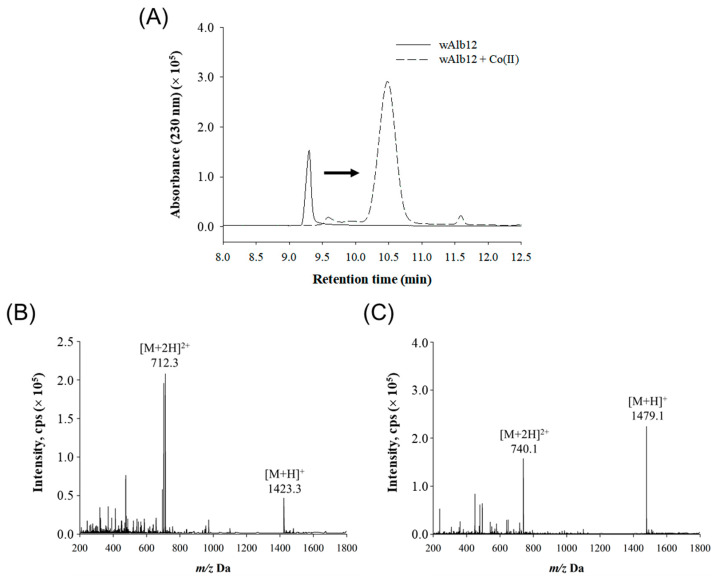
The LC-MS analysis and molecular weights of wAlb12 and wAlb12 + Co(II). (**A**) The HPLC chromatogram of wAlb12 and wAlb12 + Co(II), (**B**) ESI-MS spectrum of wAlb12, and (**C**) ESI-MS spectrum of wAlb12 + Co(II).

Co(II) binding to wAlb12 was confirmed by evaluating the change in the retention time and molecular weight during the LC-MS analysis (Figure 5). The samples comprised 0.5 mM wAlb12 alone and 0.5 mM wAlb12 mixed with 0.5 mM CoCl_2_ at a 1:1 ratio. As observed with HPLC, the retention time of wAlb12 was approximately 9.3 min and the wAlb12 + Co(II) mixture migrated to the right with a retention time of 10.5 min. Moreover, under this condition, wAlb12 + Co(II) tended to show an approximately two-fold increase in absorbance and an increased peak area (Figure 5A). ESI-MS analysis of wAlb12 alone showed the expected values, namely 1423.3 *m*/*z* corresponding to [M + H]^+^ and 712.3 *m*/*z* corresponding to [M + 2H]^2+^ (Figure 5B). After the addition of CoCl_2_, wAlb12 + Co(II) exhibited increased *m*/*z* values, namely 1479.1 *m*/*z* corresponding to [M + H]^+^ and 740.1 *m*/*z* corresponding to [M + 2H]^2+^ (Figure 5C). Accordingly, when Co(II) was bound to wAlb12, the molecular weight increased to approximately 56 with the loss of two protons. An increased molecular weight of the complex resulted in a lower Co(II) molecular weight as two hydrogen ions were removed from the peptide for binding [28]. These results indicate that Co(II) bound to wAlb12, which resulted in increased mass spectrum values for wAlb12.

### 2.6. Binding Affinity between wAlb12 and Co(II)

**Figure 6:**
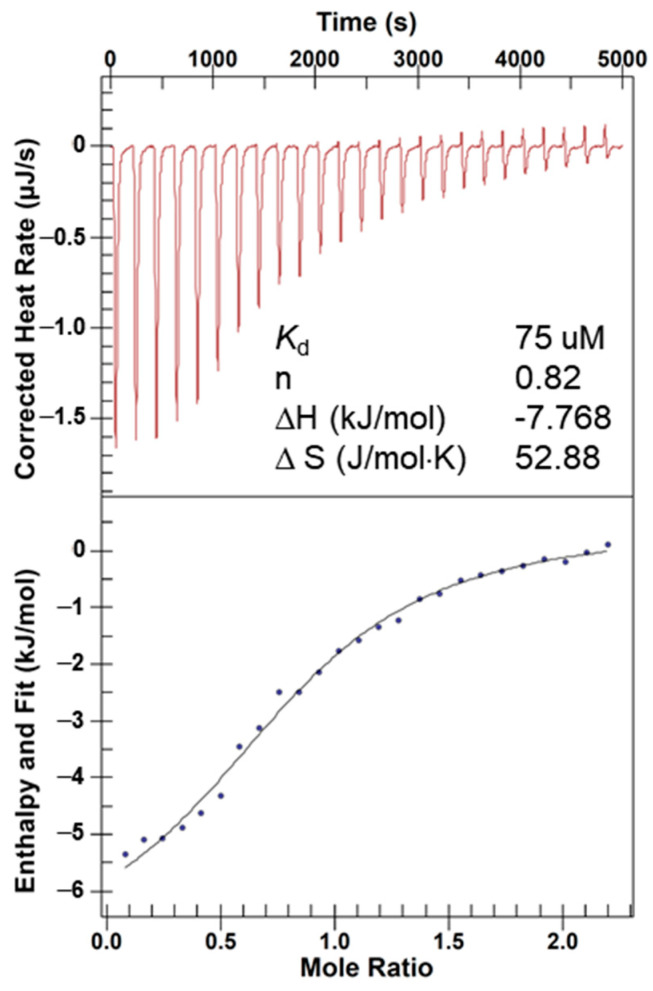
Measurement of binding affinity of the wAlb12 to Co(II) by isothermal titration calorimetry (ITC). The ITC analysis was performed three times and a representative titration curve was shown.

The binding affinity between wAlb12 and Co(II) was determined by ITC. The wAlb12 bound Co(II) with *K*_d_ of 75 μM when analyzed by a single-site binding model. The stoichiometry of the interaction was 0.82 (n) (Figure 6). In previous reports, the dissociation constant for Co(II) ion binding to the N-terminal sequence (NTS) of HSA was 110 μM [35,36], which is similar to our data. For other metal ions, the dissociation constants for Cu(II) and Ni(II) binding to the NTS of HSA were 1 pM and 150 nM, respectively. These indicate that Co(II) is a relatively low-affinity transition metal ion to the HSA. We could not obtain appropriate *K*_d_ values at different pH and temperature conditions because the heat changes were uneven and weak (Figure S3).

### 2.7. Concentration-Dependent Titration of wAlb12-Co(II) Binding Using RP-HPLC

The binding ratio and intensity based on the Co(II) concentrations were determined by RP-HPLC. When Co(II) was added at a 1:2 ratio, there was a decrease in the peak area of 0.5 mM wAlb12 (Figure S4). The results show that the Co(II) concentration was less than 0.5 mM and only the wAlb12 peaks decreased continuously, while the wAlb12 + Co(II) peaks gradually increased. Additionally, when the Co(II) concentration was 0.5 mM or higher, the wAlb12 peaks were no longer visible. Therefore, we concluded that Co(II) and wAlb12 were bound at a ratio of 1:1. The relative peak area of wAlb12 + Co(II) could not be observed above 0.5 mM (Figure S4). It has been reported that Co(II) binds to the DAHK sequence [28], and that the peak of the natural form of wAlb12 decreases up to 0.5 mM (1:1). Therefore, we considered that one Co(II) binds to one peptide.

### 2.8. Alanine Scanning for the Identification of the Co(II) Binding Site

The analysis of the binding properties of the alanine mutants was carried out using LC-MS. All of the analogs were mixed with 0.5 mM CoCl_2_ at a concentration of 0.5 mM, and the changes in the molecular weight and retention time were determined. The D1A, A2S, K4A, S5A, E6A, V7A, A8S, H9A, R10A, F11A, and K12A analogs showed that a 0.5–1 min shift in the retention time increased the molecular weight to approximately 56 (Figure S5). D1A showed a value of 1378.9 *m*/*z* and D1A + Co(II) gave the value of 1435.7 *m*/*z* at approximately 11.3 min. A2S showed a value of 1440.1 *m*/*z*, and when bound to Co(II), it showed a value of 1495.9 *m*/*z*. Likewise, the molecular weight of K4A, S5A, E6A, V7A, A8S, H9A, R10A, F11A, and K12A increased the MS values from 1366.2 to 1423.1, 1407.4 to 1463.2, 1365.2 to 1421.2, 1394.9 to 1450.6, 1440.0 to 1495.4, 1357.5 to 1413.0, 1338.1 to 1393.9, 1346.9 to 1403.2, and 1366.2 to 1422.1 *m*/*z*, respectively, which was a change of about 56 in each case. However, the Ac-wAlb12 and H3A analogs were unchanged. Thus, these results indicate that the histidine residue and N-terminal α-amino group are related to binding with Co(II) (Table 2). These results are consistent with the previously reported data [28]. The alanine scanning of almost all of the wAlb12 analogs showed that they bound to Co(II). Using LC-MS, we confirmed that the Ac-wAlb12 and H3A analogs were not bound to Co(II) (Figure S5). Thus, it was confirmed that the histidine and N-terminus of wAlb12 are essential for designing metal-binding peptides. Using the above results, we modeled the wAlb12 and Co(II) interaction, considering the binding of Co(II) to the N-terminal amide residue of D1, amide bond of D1 and A2, amide bond of A2 and H3, nitrogen of the imidazole ring of H3, and two water molecules as an octahedron (Figure S6).

### 2.9. Stability of wAlb12 and wAlb12 + Co(II) against Trypsin

**Figure 7:**
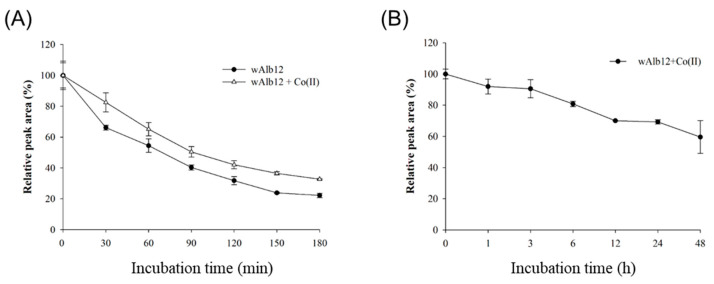
Stability of wAlb12+Co(II). Proteolytic stability of wAlb12 and wAlb12 + Co(II) complex (**A**) and human serum stability of wAlb12+Co(II) complex in vitro (**B**).

To confirm the proteolytic stability of wAlb12 and wAlb12 + Co(II), the peptides were incubated with trypsin for 3 h at 37 °C, following which HPLC was performed (Figure 7A). In the case of wAlb12, up to 66% of the peptides were not degraded after 30 min. In the case of wAlb12 + Co(II), 82% of the complex was not degraded after 30 min. At 180 min, up to 22% of wAlb12 remained; however, with wAlb12 + Co(II), 33% of the complex remained because of the binding of the peptide to Co(II). As Co(II) was bound to wAlb12, wAlb12 degraded slowly, and the stability of this complex was approximately 10% or higher compared to wAlb12. The results showed that wAlb12 + Co(II) had increased proteolytic stability. The structural change of wAlb12 after binding to Co(II) made it difficult for trypsin to selectively interact with it, resulting in lower levels of degradation than those observed for wAlb12.

### 2.10. Stability of wAlb12 and wAlb12 + Co(II) in Human Serum

The stability of the compounds in human serum was analyzed using RP-HPLC. wAlb12 + Co(II) was incubated in human serum at 37 °C for 48 h. We found that the peak area of the wAlb12 + Co(II) complex slowly decreased with the passage of time (Figure 7B). For wAlb12 + Co(II), the Co(II) was separated by approximately 10% after 1 h. Then, after 6 h, the peak area of wAlb12 + Co(II) decreased by 80%. In addition, the peak area was reduced by about 70% in 24 h, and by approximately 59% after 48 h. From these results, we suggest that the proteolytic and serum stability of wAlb12 must be improved through peptide sequence modification.

### 2.11. Cell Viability Analysis of wAlb12 and wAlb12 + Co(II)

**Figure 8:**
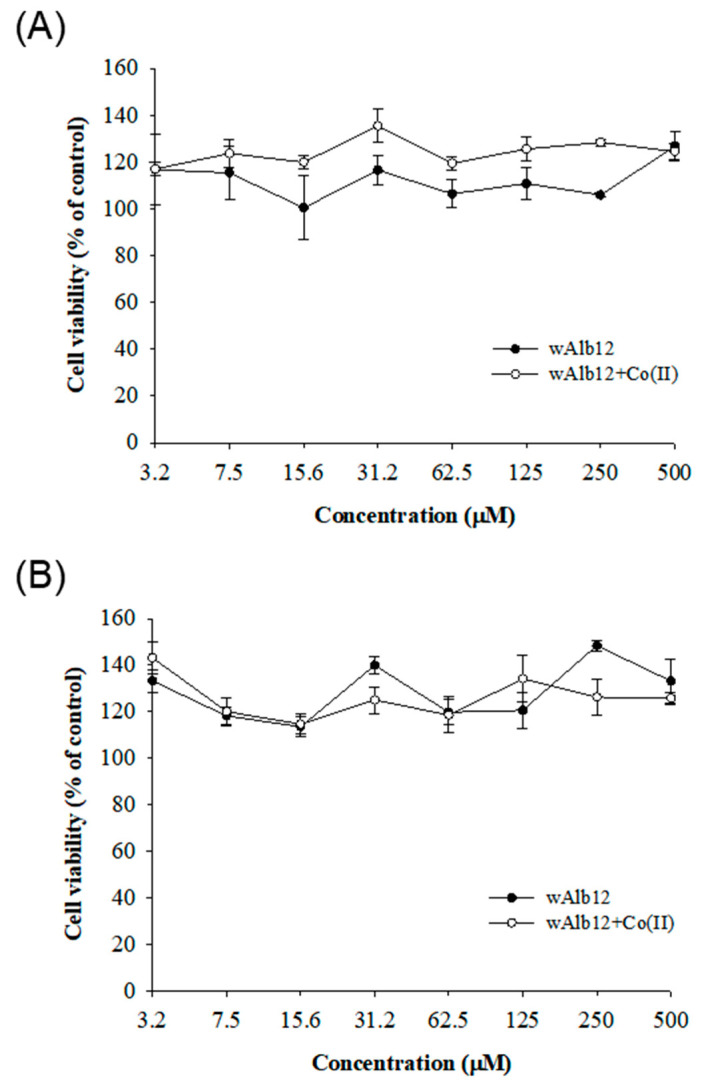
The cell viability of the wAlb12 and wAlb12 + Co(II) complex. Cell viability in RAW 264.7 cell (**A**) and in HEK 293t cell (**B**).

For use in drugs or diagnostic agents administered to humans, the wAlb12 + Co(II) complex must be nontoxic to host cells. Therefore, we assessed the cytotoxicity of wAlb12 and wAlb12 + Co(II) in vitro (Figure 8). wAlb12 and wAlb12 + Co(II) were not cytotoxic to RAW 264.7 cells at 0.5 mM concentrations (Figure 8A). The wAlb12- and wAlb12 + Co(II)-treated cells displayed increased cell viability compared to those in the control medium. Thus, the cell viability constant was maintained at more than 100% at all concentrations. Likewise, the two compounds were also non-toxic to HEK 293T cells (Figure 8B). The results showed that wAlb12 and wAlb12 + Co(II) are neither toxic to RAW 264.7 or HEK 293T cells.

## 3. Materials and Methods

All amino acids for peptide synthesis were purchased from the GL Biochem (Shanghai, China), and cobalt chloride (CoCl_2_) was purchased from Alfa-Aesar (Carlsbad, CA, USA). Trypsin and human serum were purchased form Gibco and Sigma–Aldrich, respectively. For the cell culture, Dulbecco’s modified Eagle’s medium (DMEM) (Gibco, Fort Worth, TX, USA), fetal bovine serum (FBS) (Capricorn, Ebsdorfergrund, Germany), and penicillin-streptomycin (P/S, Gibco) were purchased. Chemicals and reagents were of analytical grade and were supplied by Sigma–Aldrich (St. Louis, MO, USA).

### 3.1. Peptide Synthesis

All of the peptides used in this experiment were synthesized by solid phase peptide synthesis (SPPS), as performed previously [37]. To explain briefly, amide resin was treated with 20% piperidine to remove the Fmoc protecting group. After washing three times with dichloromethane (DCM) and dimethylformamide (DMF), amino acids dissolved in DMF and coupling reagents (2 M hydroxybenzotriazole and 2 M N,N′-diisopropylcarbodiimide) were added. After 3 h of reaction, this process was repeated to synthesize the entire sequence of the peptide. To cleave the completely synthesized peptide from the resin and the protecting functional group of amino acids, the synthesized peptides were treated with the cleavage solution (82.5% trifluoroacetic acid (TFA), 5% H_2_O, 2.5% ethanedithiol, 5% phenol, and 5% thioanisole) and allowed to react for 3 h at room temperature. The cleaved peptides were precipitated by centrifuging at 12,000× *g* for 3 min using eight times the volume of cold diethyl ether. All precipitation experiments after cleavage were performed on ice. The precipitated peptides were purified by reverse phase high pressure liquid chromatography (LC) (RP-HPLC, LC-6AD, Shimadzu, Kyoto, Japan) using the Shim-Pack C18 column (20 × 250 mm, Shimadzu) at 35 °C. The mobile phases of the RP-HPLC were buffer A (100% water containing 0.05% TFA) and buffer B (100% acetonitrile containing 0.05% TFA). Next, the purity of the peptides was confirmed using analytical LC (RP-HPLC, LC-10AD, Shimadzu)-MS (API2000, AB SCIEX, Redwood, CA, USA). Finally, the purified peptides were lyophilized for further experiments.

### 3.2. Alanine Scanning Mutagenesis

A mutation was performed to identify the binding properties of wAlb12. Twelve amino acids of wAlb12 were substituted with alanine and the remaining alanine of wAlb12 was substituted with serine (D1A, A2S, H3A, K4A, S5A, E6A, V7A, A8S, H9A, R10A, F11A, and K12A). In addition, the N-terminal region of wAlb12 was acetylated (Ac-wAlb12). The only N-terminal α-amino group of wAlb12 was acetylated under 70% DCM, 25% acetic anhydride, and 5% triethylamine for 2 h after Fmoc cleavage by 20% piperidine. All of the analogs were synthesized by SPPS, and the purity and molecular weight were confirmed using liquid chromatography-mass spectrometry (LC-MS). The high-performance liquid chromatography (HPLC) gradient program was set up with 5% to 35% buffer B for 15 min, and the molecular weights of the prepared analogs were confirmed using MS in the range of 200 to 1800 *m*/*z*.

### 3.3. UV Spectrophotometry

UV-visible spectroscopy (UV-1650 PC, Shimadzu) was used to investigate the Co(II)-binding properties of the wAlb12 and SMAP18 (G2–G13) peptides. The purified peptides and CoCl_2_ were prepared in 50 mM sodium phosphate buffer with 10 mM sodium chloride buffer solution. The wAlb12 and SMAP18 (G2–G13) peptides (0.5 mM) were mixed with up to 1 mM (1:2 ratio) of CoCl_2_. Then, after 30 s of vortexing, the mixture was placed in a disposable quartz cuvette and the absorbance was measured at wavelength ranges of λ = 230–360 nm.

### 3.4. Circular Dichroism (CD) Spectroscopy

CD spectroscopy was carried out using the J-810 spectropolarimeter (JASCO, Japan) and a cell path length of 1.0 mm at 25 °C. The wAlb12 and SMAP18 (G2–G13) peptides were dissolved in 50 mM sodium phosphate buffer with 10 mM sodium·chloride, and the concentration of the peptides was 0.1 mM. The peptides were reacted with 0.05 (1:0.5), 0.1 (1:1), and 0.2 mM (1:2) of CoCl_2_. Time scans of the CD spectra were recorded three times from 190 to 260 nm with sampling points of 1 nm. A volume of 400 µL was used for measurement and placed in a rectangular quartz cell. The data were converted to molar ellipticity, [θ]_mr_ (deg cm^2^.dmol^−1^). The mean of the ellipticity, [θ]_mr_ (deg.cm^2^.dmol^−1^), was converted by the following equation:

θ M = (θ obs × 1000)/(c × ℓ × n)

where θ M, θ obs, c, ℓ, and n are the centimeter per decimole, scan value, peptide concentration, path length, and number of amino acids, respectively.

### 3.5. NMR Spectroscopy

The 1D proton NMR spectra were measured at 298K using an NMR spectrometer (AVANCE 600 MHz, Bruker, Germany) to investigate signal changes of wAlb12 according to the presence or absence of Co(II). The NMR data were processed using the MNOVA software package. The wAlb12 peptide and CoCl_2_ were dissolved in 50 mM sodium phosphate with 10 mM NaCl buffer (pH 7.4), and the NMR samples were prepared with 10% D_2_O in 0.25, 0.5, and 1 mM of CoCl_2_.

### 3.6. HPLC and LC-MS Analysis

To verify the binding of peptides to Co(II), HPLC and LC-MS were performed to confirm the retention time and molecular weight. RT-HPLC analyses were performed using Shimadzu 10AD system with a Sunfire^®^ column (C18 5 μm 4.6 mm × 250 mm, Waters, Milford, MA, USA) and a flow rate of 1 mL/min with buffer B (100% acetonitrile containing 0.05% TFA) and buffer A (100% H_2_O containing 0.05% TFA). The eluted components were monitored at an absorbance of 230 nm with a gradient from 5% to 35% of buffer B for 15 min. Then, the molecular weight of the peptides was confirmed using a LC-MS spectrometer with ESI electrospray (AB Sciex API2000, Redwood, CA, USA).

### 3.7. Isothermal Titration Calorimetry

The wAlb12–Co(II) interaction was analyzed by isothermal titration calorimetry (ITC) using an Affinity ITC calorimeter (TA instruments, New Castle, DE, USA) at 25 °C. All of the samples were prepared in the identical buffer containing 50 mM sodium phosphate with 10 mM NaCl. The syringe was loaded with 5 mM of the CoCl_2_ and the cell was filled with 350 μL of 0.5 mM wAlb12. The titration curve was obtained by injecting 2 μL  ×  25 aliquots of the CoCl_2_ into the cell at a time interval of 300 s. The enthalpy of the reaction, ΔH0, the dissociation constant, *K*_d_, and the stoichiometry value, n, were calculated from the measured heat changes, δH_i_, upon the association of the wAlb12 and CoCl_2_. The titration data were analyzed using the NanoAnalyze program (TA instruments) and fitted into a single-site binding model.

### 3.8. Stability

#### 3.8.1. Protease Stability

The protease stability of wAlb12 and wAlb12 + Co(II) was measured based on the relative peak area obtained by RP-HPLC at a wavelength of 230 nm as described by Meng and Kumar [38] with a few modifications. wAlb12 and wAlb12 + Co(II) (0.6 mg/mL) were treated with trypsin (0.6 μg/mL) at a molar ratio of 1000:1 in 50 mM sodium phosphate with 10 mM sodium chloride buffer solution (pH 7.4) at 37 °C for 3 h. TFA (2.5%, 20 μL) was added to 80-μL aliquots of this mixture to inhibit the trypsin activity and then vortexed for 30 s. The mixtures were filtered using the Spin-X Column (0.22 μm, COSTAR, Washington, DC, USA). Then, 40 μL of each of the filtered samples was injected into the RP-HPLC system. All of the experiments were performed in triplicate.

#### 3.8.2. Serum Stability

The serum stability of Co(II)-bound wAlb12 was assessed based on the method of Nguyen, Chau [39], with a few modifications. wAlb12 + Co(II) (1 mM) and human serum were mixed with 700 µL at a ratio of 1:1. Then, the mixture was vortexed for 1 min and incubated at 37 °C. At the specified time point, 50 µL of the mixture was transferred to a new 1.5-mL microtube and 50 µL of buffer B was added, followed by centrifugation at 15,000× *g* for 3 min. Next, the supernatant was transferred to a 1.5-mL tube and 100 µL of buffer A was added. Finally, the samples were analyzed using RP-HPLC after filtration using the Spin-X Column. The samples were injected at 100 µL, and all of the experiments were performed in triplicate.

### 3.9. Cell Culture Preparation

RAW 264.7 and HEK 293T cells were cultured at 37 °C under 5% CO_2_ in 95% DMEM supplemented with 10% FBS and 1% P/S antibiotics. The RAW 264.7 and HEK 293T cells were harvested in 3 mL of DMEM, and centrifugation was performed at 1200× *g* using the AllegraTM X-12R centrifuge (BECKMAN COULTER). The cell pellet was resuspended in 1 mL of DMEM. The number of cells was counted using a hemocytometer.

### 3.10. Cell Viability Assay

The cell viability was measured using MTT assay according to the method of Mirzapour-Kouhdasht, Moosavi-Nasab [40], with a few modifications. The cells were first plated at a density of a 5 × 10^4^ cells/well in a 96-well plate. After overnight incubation, the wAlb12 and wAlb12 + Co(II) each dissolved in buffer were used to treat the cells by concentration, which were then incubated for 24 h at 37 °C. Next, 10 µL of 5 mg mL^−1^ MTT stock solution was added to each well, and the plate was incubated for 4 h at 37 °C. Next, the medium was removed and 100 µL of DMSO was added to each well to lyse the cells. The treated cells were incubated at 37 °C for 20 min and their absorbance was measured at a wavelength of 600 nm using an ELISA reader (Autobio, Phomo). The percentage viability was calculated using the following formula:

Cell viability (%) = [(As) − (Ab)]/[(Ac) − (Ab)] × 100

where As, Ab, and Ac are the absorbance values of the peptide-treated cells, blank, and control samples, respectively.

### 3.11. Statistical Analysis

The data obtained from triplicate experiments were analyzed by one-way ANOVA using IBM SPSS version 25, at a confidence level of 95% using Tukey’s test.

## 4. Conclusions

In this study, we designed wAlb12 bound to Co(II) and evaluated its properties, intensity, and the binding site for use as a chelating agent. After binding with Co(II), the structure of wAlb12 changed and the chemical shifts were represented. Additionally, using LC-MS, we confirmed the increased absorbance intensity and molecular weight. The Co(II) self-assembled with wAlb12 showed strong stability at neutral or basic pH and different temperatures. The binding of the peptide to Co(II) resulted in increased stability against proteolytic digestion. Furthermore, we confirmed that there was no cytotoxicity in rat and human cell lines in vitro. The H3A analog, which substituted histidine with alanine, and acetylated wAlb12 did not bind to Co(II). Thus, the N-terminus of the α-amino group and 3-histidine are essential sites that interact with Co(II). Therefore, the use of wAlb12 as a peptide-based radioactive isotope chelating agent after modifying to improve binding affinity and stability is suggested.

## Figures and Tables

**Table 1:** Sequences, molecular weights, and detected MS values of the synthesized peptides.

Number	Peptide	Sequence	Theoretical Mass	Detected [M + H]^+^	Detected [M + 2H]^2+^
1	wAlb12	DAHKSEVAHRFK	1423.58	1424.6	713.3
2	Poly(his)8	HHHHHHHH	1114.14	1115.3	558.3
3	SMAP18 (G12–G13)	GLRRLGRKIAHG	1332.61	1333.3	667.2
4	D1A	AAHKSEVAHRFK	1379.57	1378.9	690.3
5	A2S	DSHKSEVAHRFK	1439.58	1440.1	720.0
6	H3A	DAAKSEVAHRFK	1357.52	1357.1	680.7
7	K4A	DAHASEVAHRFK	1366.49	1366.0	683.9
8	S5A	DAHKAEVAHRFK	1407.58	1408.3	704.1
9	E6A	DAHKSAVAHRFK	1365.55	1365.1	683.5
10	V7A	DAHKSEAAHRFK	1395.53	1394.9	698.7
11	A8S	DAHKSEVSHRFK	1439.58	1440.0	719.8
12	H9A	DAHKSEVAARFK	1357.52	1358.3	679.1
13	R10A	DAHKSEVAHAFK	1338.47	1337.1	661.3
14	F11A	DAHKSEVAHRAK	1347.48	1347.0	674.2
15	K12A	DAHKSEVAHRFA	1366.49	1366.3	683.9
16	Ac-wAlb12	Ac-DAHKSEVAHRFK	1464.58	1465.0	733.2

**Table 2:** MS and analysis of cobalt binding with alanine scanning analogs.

Peptide	Detected [M + H]^+^	Adding Co(II)		
		Detected [M + H]^+^	Retention Time (min)	Binding
D1A	1378.9	1435.7	9.5 → ^1^ 11.4	Yes
A2S	1440.1	1495.9	9.0 → 11.0	Yes
H3A	1357.1	1357.1	10.5	No
K4A	1366.0	1423.1	9.2 → 10.0	Yes
S5A	1408.3	1463.2	9.5 → 11.5	Yes
E6A	1365.1	1421.2	9.6 → 11.0	Yes
V7A	1394.9	1450.6	9.0 → 9.8	Yes
A8S	1440.0	1495.4	9.4 → 10.3	Yes
H9A	1358.3	1413.0	10.5 → 11.0	Yes
R10A	1337.1	1393.9	10.2 → 11.7	Yes
F11A	1347.0	1403.2	7.3 → 8.0	Yes
K12A	1366.3	1422.1	10.4 → 12.0	Yes
Ac-wAlb12	1465.0	1465.1	10.0	No

^1^ “→” means the retention time shift by cobalt binding.

## Data Availability

The data that support the findings of this study are available from the corresponding author upon reasonable request.

## Supplementary Materials

The following supporting information can be downloaded at: https://www.mdpi.com/article/10.3390/ijms23020719/s1.
