# Supplementary material for: Development of Cobalt-Binding Peptide Chelate from Human Serum Albumin: Cobalt-Binding Properties and Stability
Source: Int J Mol Sci. 2022 Jan 10;23(2):719. doi: 10.3390/ijms23020719 (PMC8775498; doi:10.3390/ijms23020719)
Supplement: Supplementary file 1 [file ijms-23-00719-s001.zip › ijms-1499975-supplementary.pdf]

International Journal of *Molecular Sciences*

MDPI

Article

Development of Cobalt-Binding Peptide Chelate from Human Serum Albumin: Cobalt-Binding Properties and Stability

Yeonje Cho [1], Armin Mirzapour-Kouhdasht [1,2], Hyosuk Yun [1], Jeong Hoon Park [3], Hye Jung Min [4] and Chul Won Lee [1,*]

1 Department of Chemistry, Chonnam National University, Gwangju 61186, Korea; yeonje20@gmail.com (Y.C.); armin.mirzapourkouhdahst@ucd.ie (A.M.-K.); 5300747yun@hanmail.net (H.Y.)
2 School of Agriculture and Food Science, University College Dublin, Belfield, 4 Dublin, D04 V1W8, Ireland
3 Accelerator Radioisotope Development Laboratory, Korea Atomic Energy Research Institute, Jeongeup-si 56212, Jeollabuk-do, Korea; parkjh@kaeri.re.kr
4 Department of Cosmetic Science, Kwangju Women's University, Gwangju 62396, Korea; sarock@kwu.ac.kr
* Correspondence: cwlee@jnu.ac.kr; Tel.: +82-62-530-3374; Fax: +82-62-530-3389

Citation: Cho, Y.; Mirzapour-Kouhdasht, A.; Yun, H.; Park, J.H.; Min, H.J.; Lee, C.W. Development of Cobalt-Binding Peptide Chelate from Human Serum Albumin: Cobalt-Binding Properties and Stability. *Int. J. Mol. Sci.* **2022**, *23*, 719. https://doi.org/10.3390/ijms23020719

Academic Editor: Alessandra Di Masi

Received: 23 November 2021
Accepted: 7 January 2022
Published: 10 January 2022

Publisher's Note: MDPI stays neutral with regard to jurisdictional claims in published maps and institutional affiliations.

Copyright: © 2022 by the authors. Licensee MDPI, Basel, Switzerland. This article is an open access article distributed under the terms and conditions of the Creative Commons Attribution (CC BY) license (http://creativecommons.org/licenses/by/4.0/).

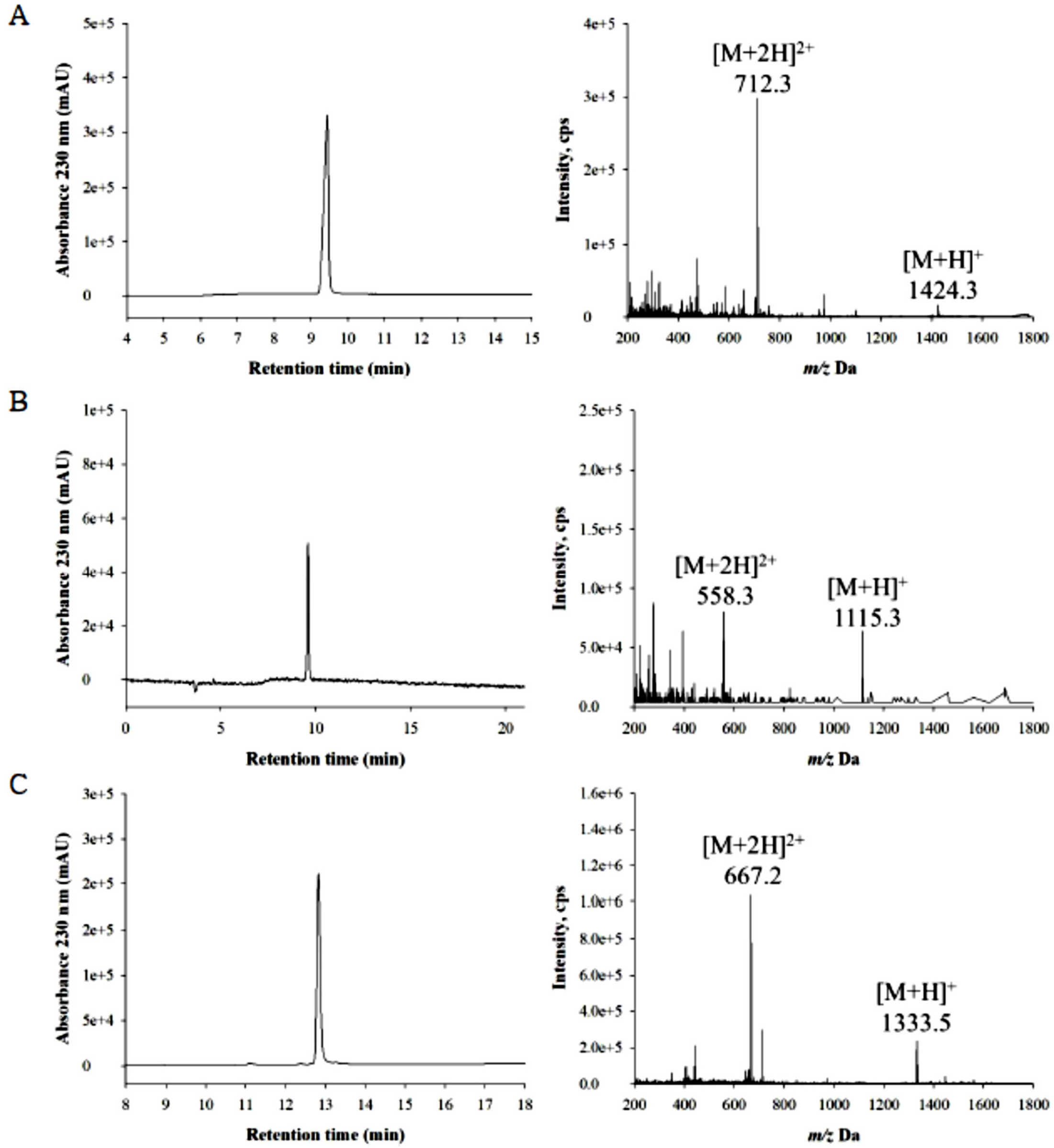

Figure S1. RP-HPLC (left) and ESI-MS spectrometric (right) analysis of the peptides. (**A**) wAlb12, (**B**) poly(his)8, and (**C**) SMAP18 (G2-G13).

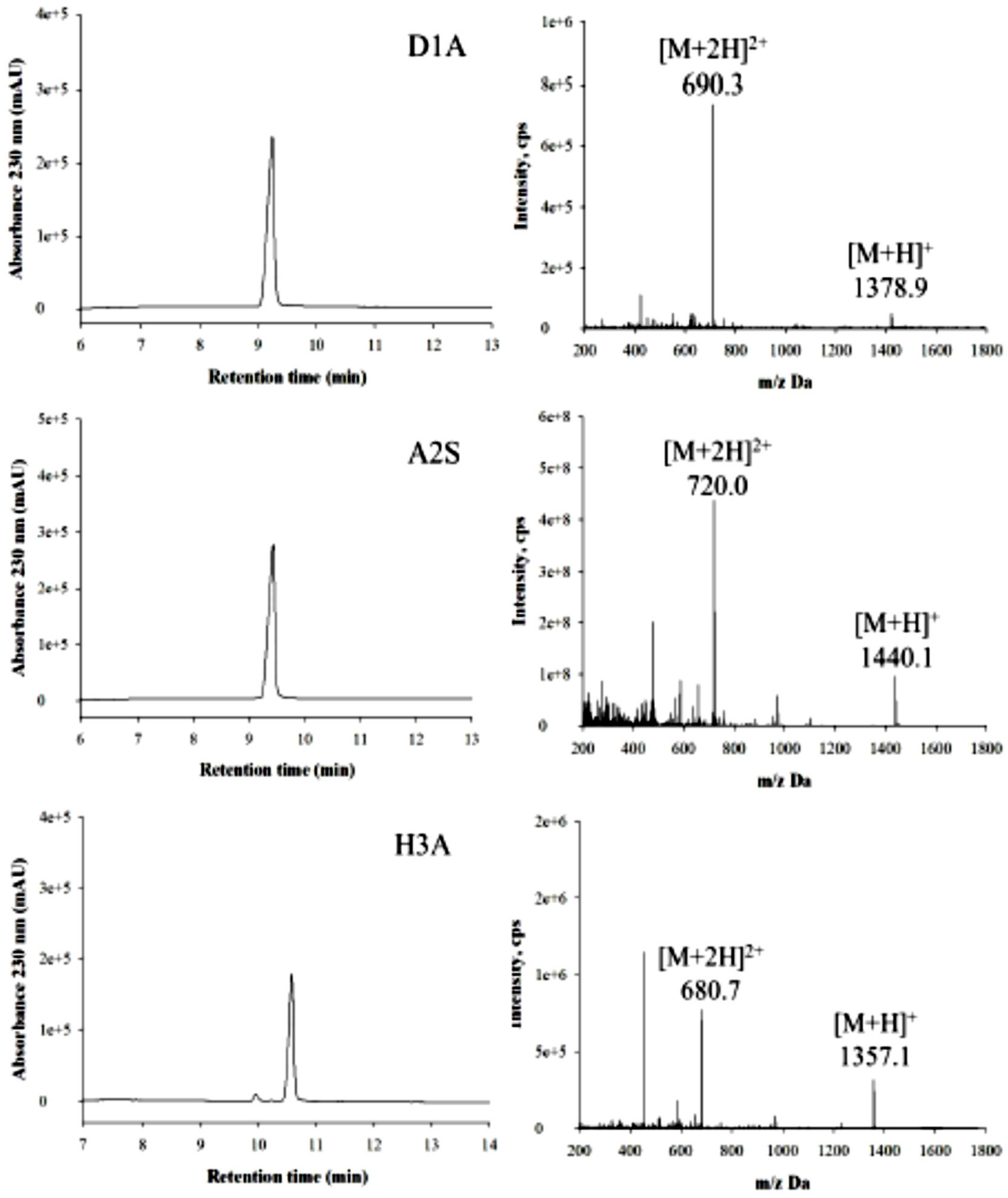
D1A
Absorbance 230 nm (mAU)
Retention time (min)
[M+2H]2+
690.3
[M+H]+
1378.9
Intensity, cps
m/z Da
A2S
[M+2H]2+
720.0
[M+H]+
1440.1
H3A
[M+2H]2+
680.7
[M+H]+
1357.1

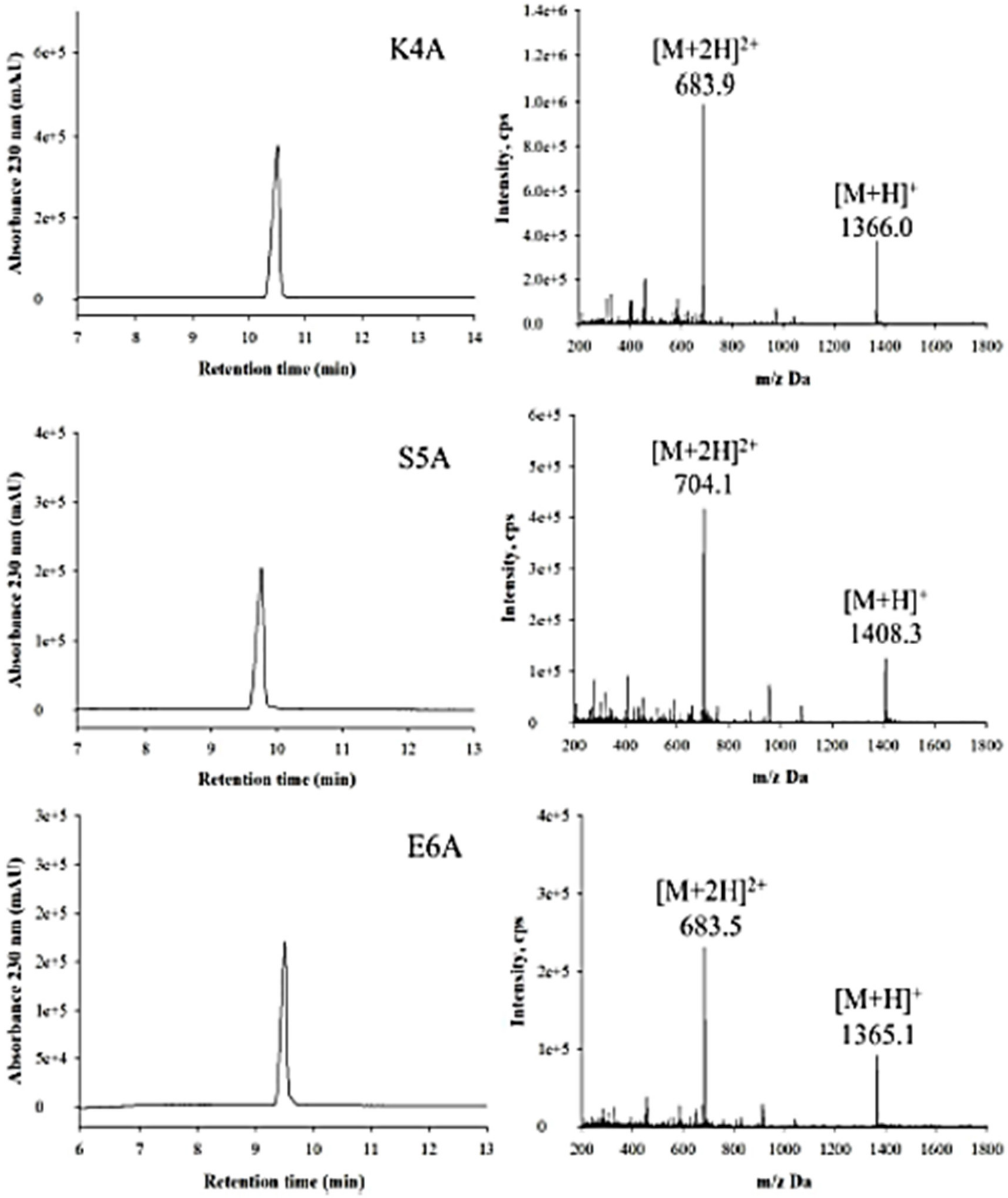

Figure S2 (*continued*)

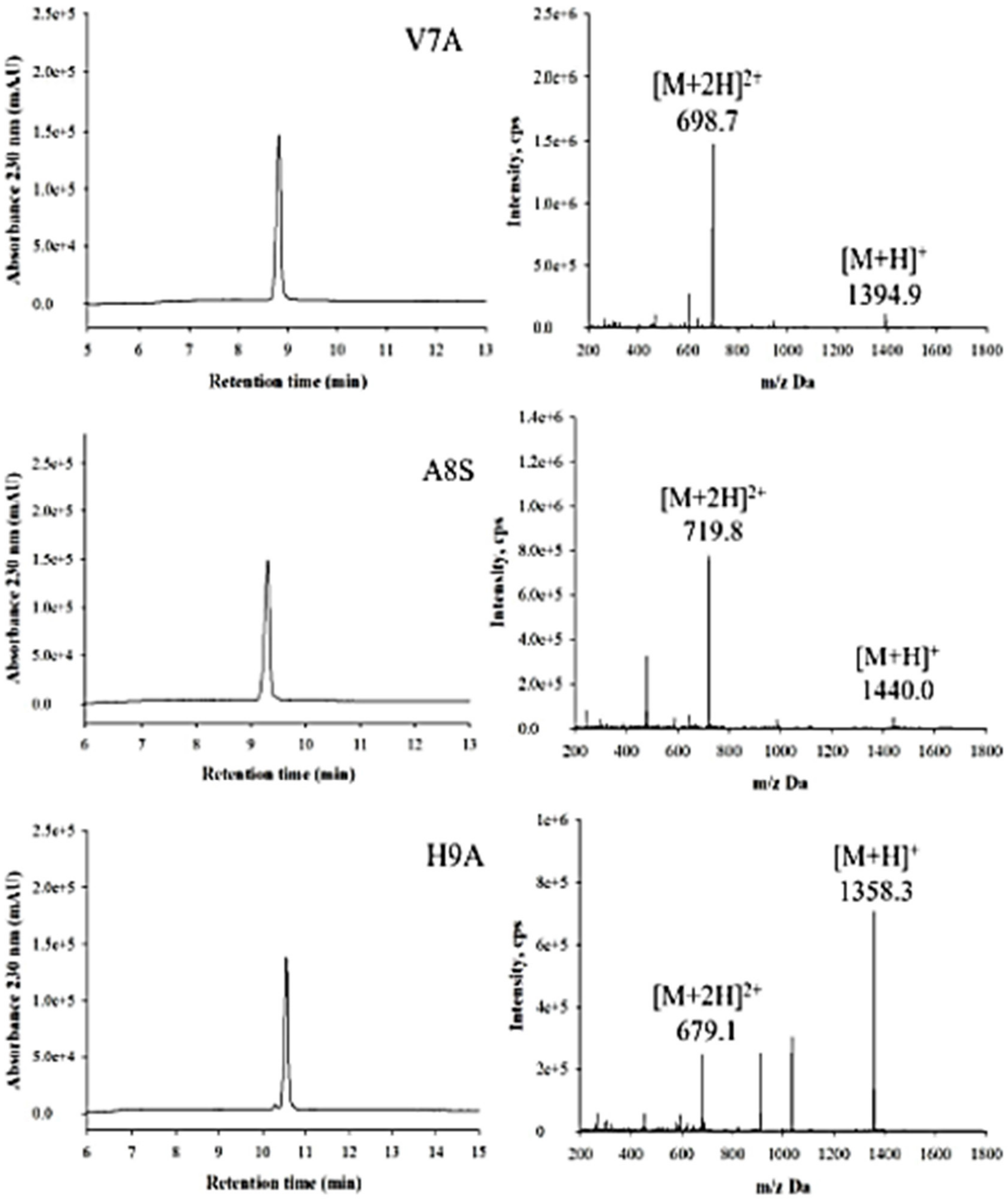

Figure S2 (*continued*)

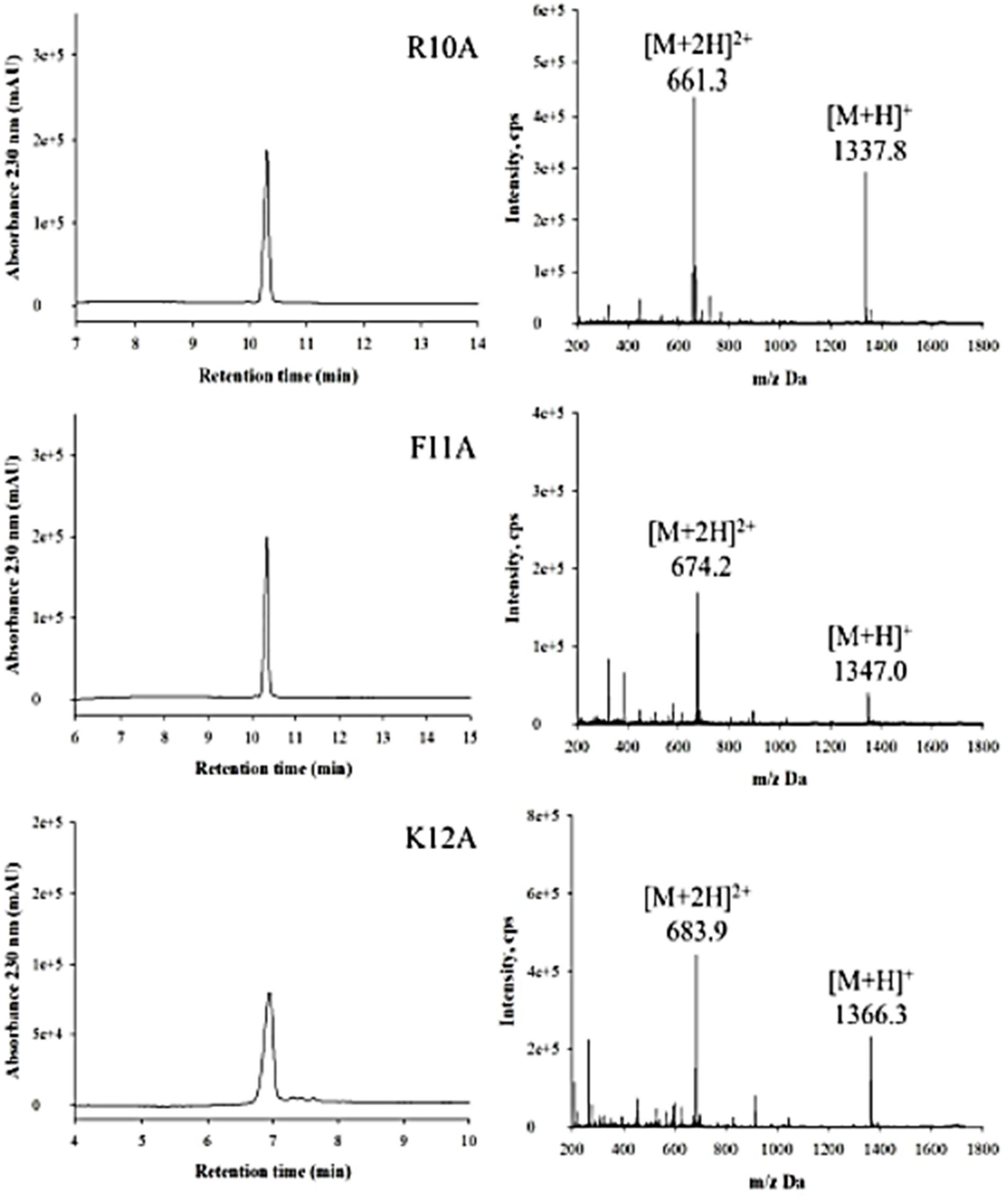

Figure S2 (*continued*)

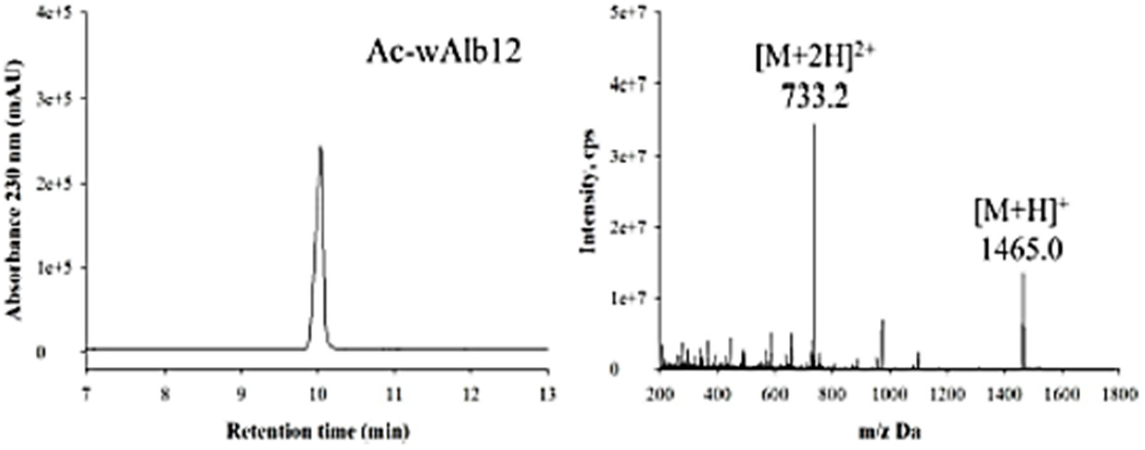

Figure S2 (*continued*)

Figure S2. RP-HPLC (left) and ESI-MS spectrometric (right) analysis of the synthesized analogues (D1A, A2S, H3A, K4A, S5A, E6A, V7A, A8S, H9A, R10A, F11A, K12A, and Ac-wAlb).

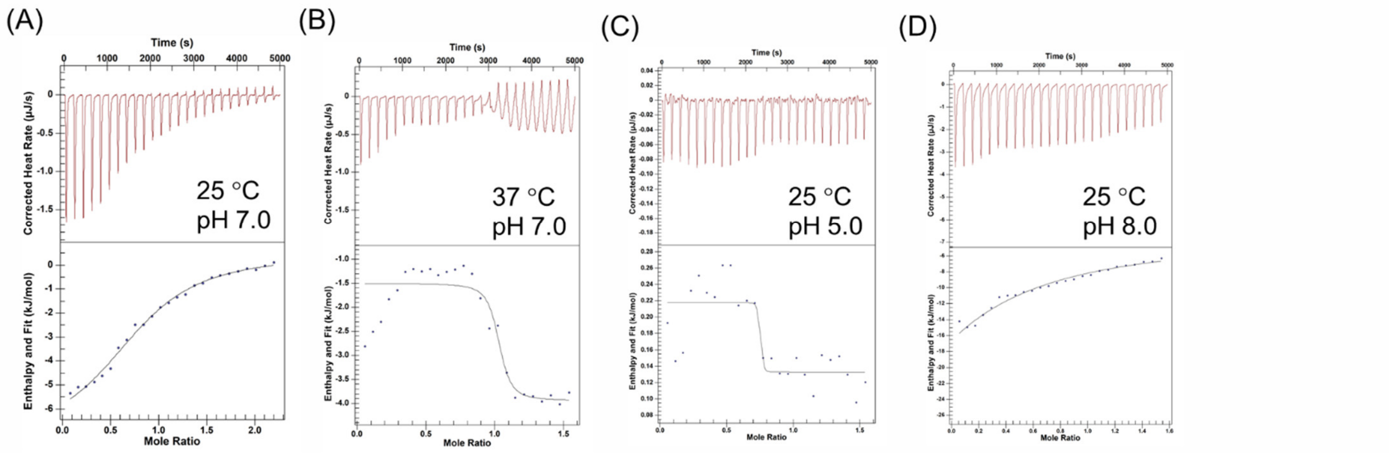

Figure S3. ITC data between wAlb12 and $CoCl_2$ at various pH and temperature. (**A**) at 25 °C and pH 7.0, (**B**) at 37 °C and pH 7.0, (**C**) at 25 °C and pH 5.0, (**D**) at 25 °C and pH 8.0.

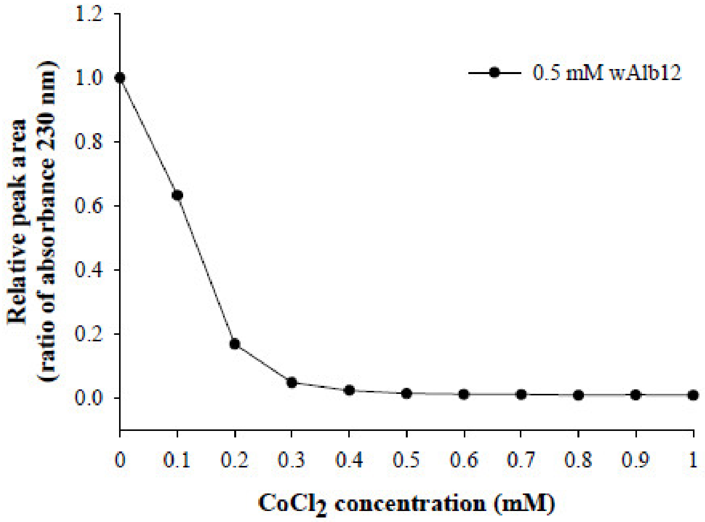

Figure S4. Concentration-dependent titration of the Co(II) binding to wAlb12.

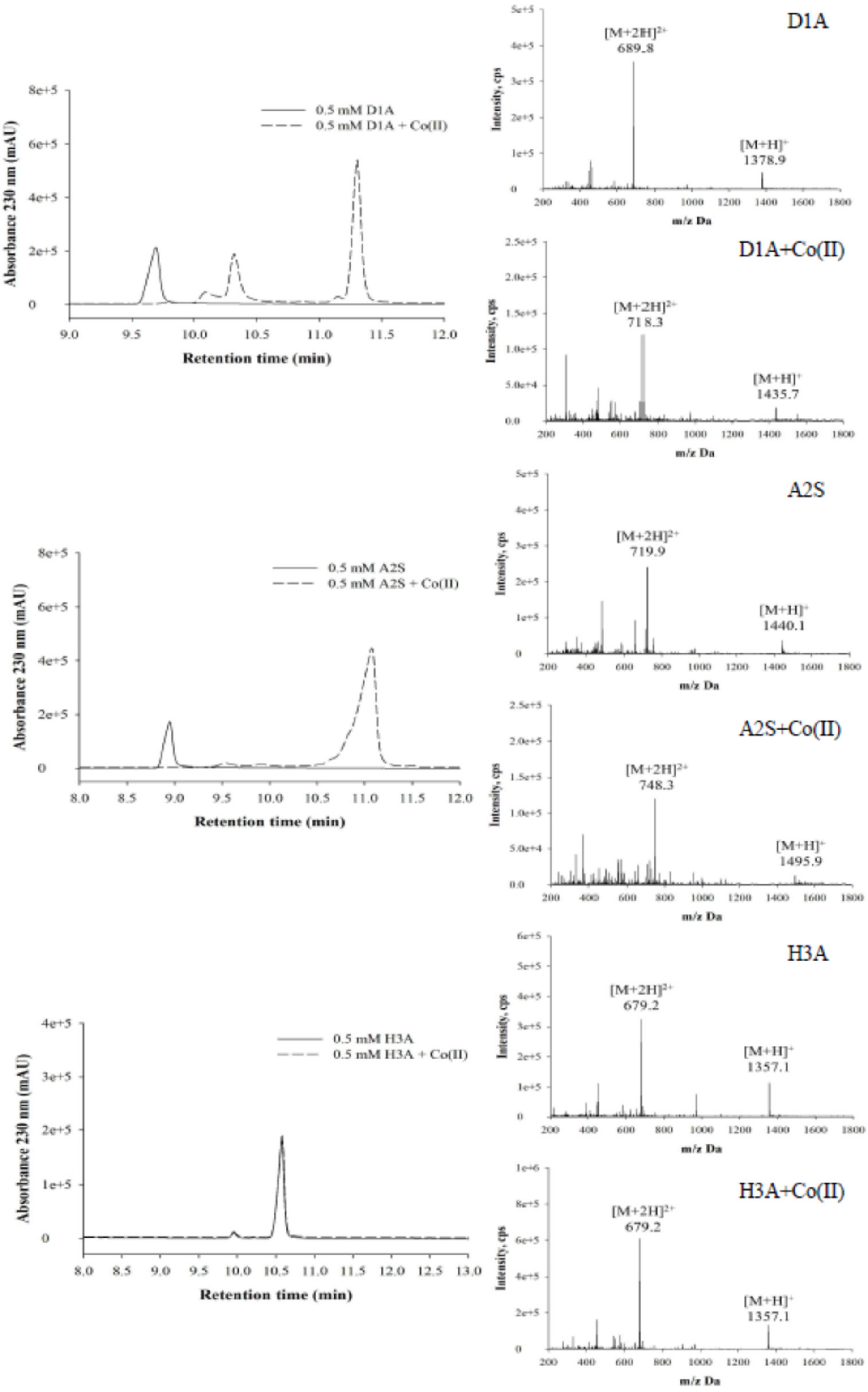
0.5 mM D1A
0.5 mM D1A + Co(II)
Absorbance 230 nm (mAU)
Retention time (min)
D1A
[M+2H]2+
689.8
[M+H]+
1378.9
D1A+Co(II)
[M+2H]2+
718.3
[M+H]+
1435.7
0.5 mM A2S
0.5 mM A2S + Co(II)
A2S
[M+2H]2+
719.9
[M+H]+
1440.1
A2S+Co(II)
[M+2H]2+
748.3
[M+H]+
1495.9
0.5 mM H3A
0.5 mM H3A + Co(II)
H3A
[M+2H]2+
679.2
[M+H]+
1357.1
H3A+Co(II)
[M+2H]2+
679.2
[M+H]+
1357.1
Intensity, cps
m/z Da

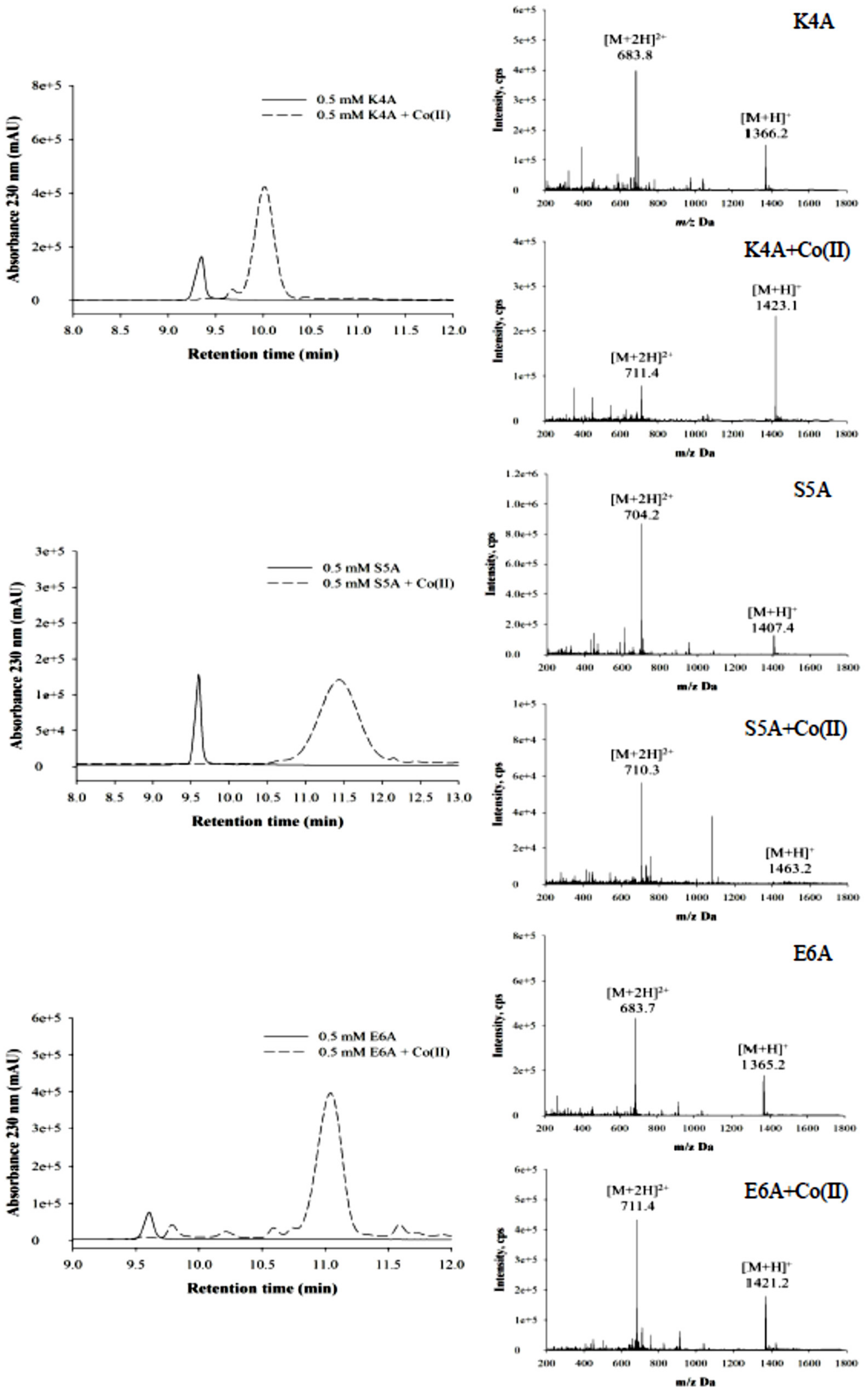

0.5 mM K4A
0.5 mM K4A + Co(II)
Absorbance 230 nm (mAU)
Retention time (min)
K4A
[M+2H]2+ 683.8
[M+H]+ 1366.2
K4A+Co(II)
[M+H]+ 1423.1
[M+2H]2+ 711.4
0.5 mM S5A
0.5 mM S5A + Co(II)
S5A
[M+2H]2+ 704.2
[M+H]+ 1407.4
S5A+Co(II)
[M+2H]2+ 710.3
[M+H]+ 1463.2
0.5 mM E6A
0.5 mM E6A + Co(II)
E6A
[M+2H]2+ 683.7
[M+H]+ 1365.2
E6A+Co(II)
[M+2H]2+ 711.4
[M+H]+ 1421.2
Intensity, cps
m/z Da

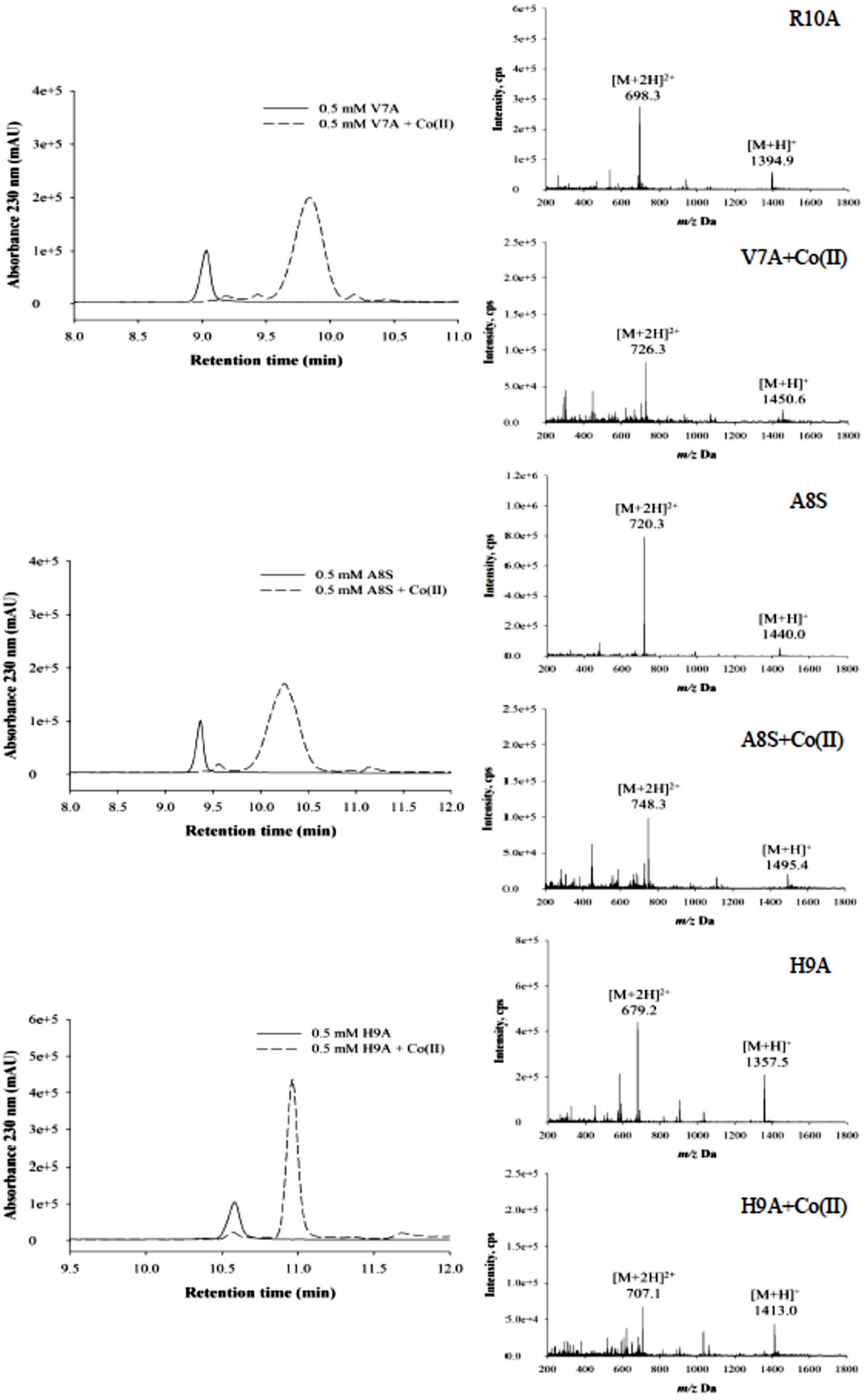
R10A
[M+2H]2+
698.3
[M+H]+
1394.9
V7A+Co(II)
[M+2H]2+
726.3
[M+H]+
1450.6
A8S
[M+2H]2+
720.3
[M+H]+
1440.0
A8S+Co(II)
[M+2H]2+
748.3
[M+H]+
1495.4
H9A
[M+2H]2+
679.2
[M+H]+
1357.5
H9A+Co(II)
[M+2H]2+
707.1
[M+H]+
1413.0
Intensity, cps
m/z Da
0.5 mM V7A
0.5 mM V7A + Co(II)
0.5 mM A8S
0.5 mM A8S + Co(II)
0.5 mM H9A
0.5 mM H9A + Co(II)
Absorbance 230 nm (mAU)
Retention time (min)

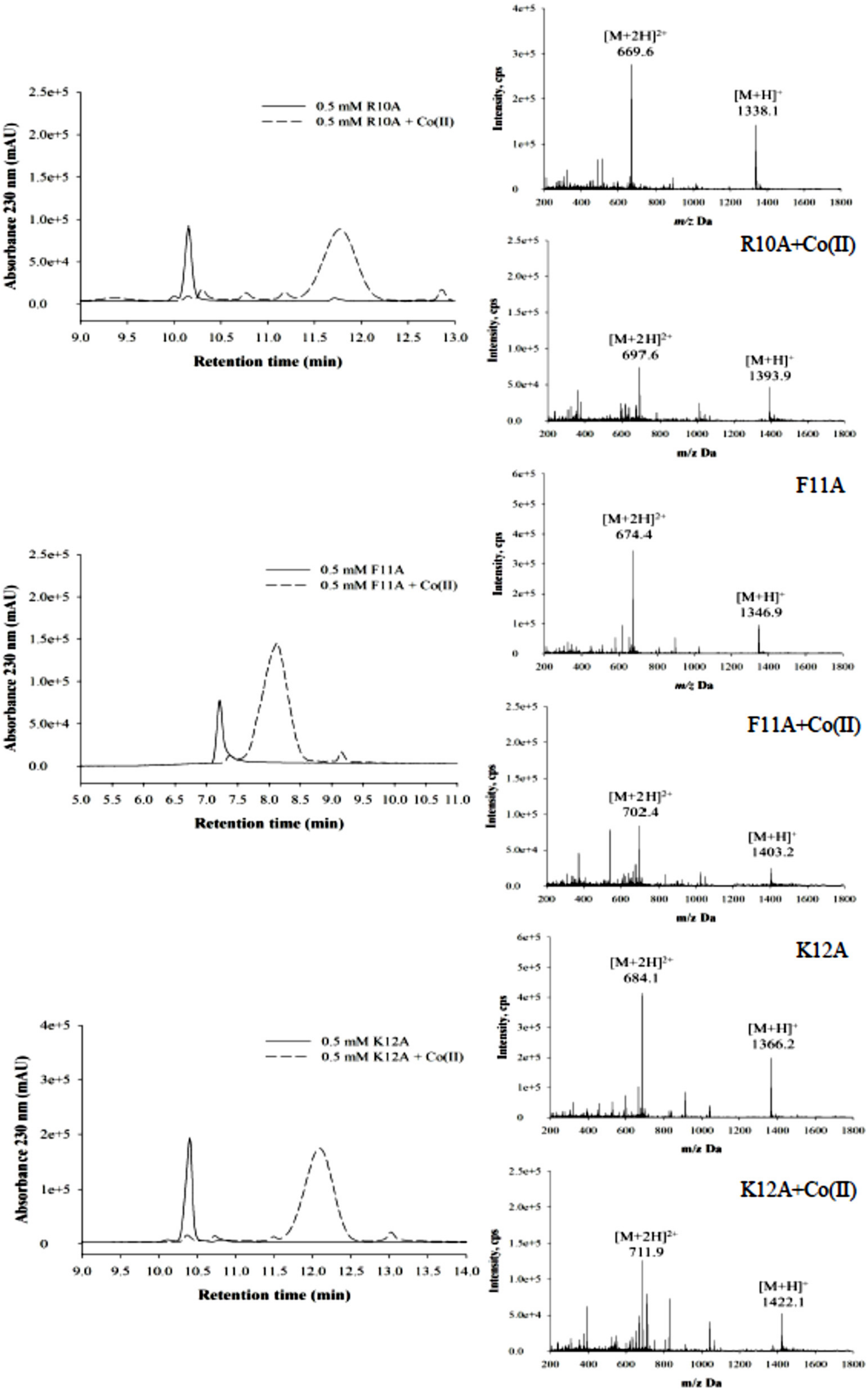
0.5 mM R10A
0.5 mM R10A + Co(II)
Absorbance 230 nm (mAU)
Retention time (min)
Intensity, cps
m/z Da
[M+2H]2+ 669.6
[M+H]+ 1338.1
R10A+Co(II)
[M+2H]2+ 697.6
[M+H]+ 1393.9
0.5 mM F11A
0.5 mM F11A + Co(II)
F11A
[M+2H]2+ 674.4
[M+H]+ 1346.9
F11A+Co(II)
[M+2H]2+ 702.4
[M+H]+ 1403.2
0.5 mM K12A
0.5 mM K12A + Co(II)
K12A
[M+2H]2+ 684.1
[M+H]+ 1366.2
K12A+Co(II)
[M+2H]2+ 711.9
[M+H]+ 1422.1

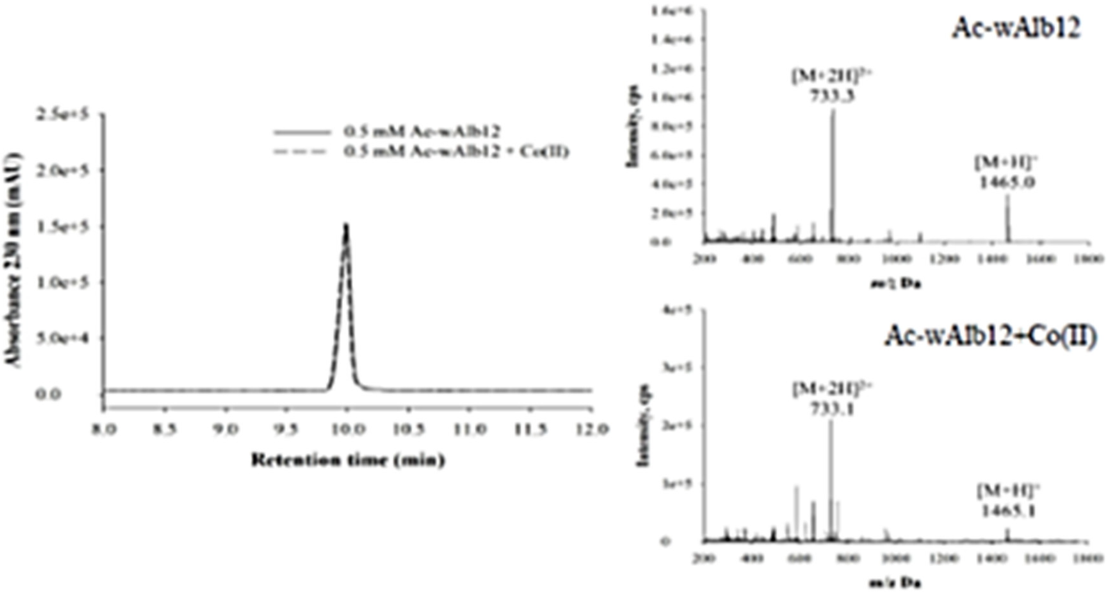

Figure S5. LC-MS analysis of alanine scanning analogs with and without Co(II). HPLC chromatogram (left) and ESI-MS spectra (right).

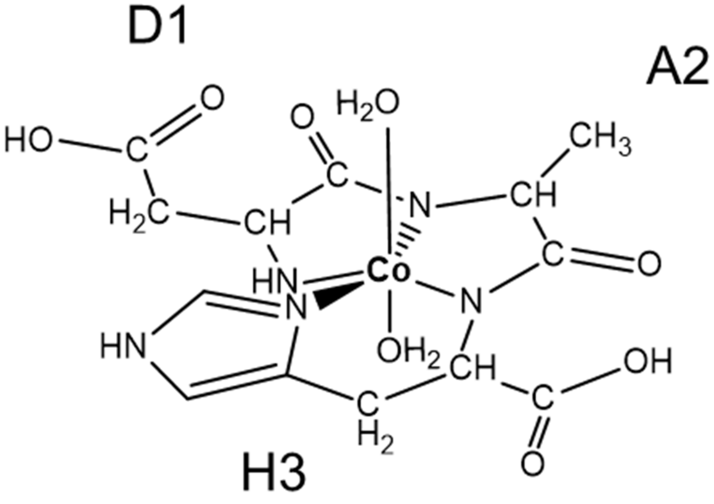

Figure S6. Model structure of the N-terminal residues of wAlb12 binding to Co(II).
